# Supplementary material for: Differential methylation at novel putative imprinting control regions on chromosome 20 associated with childhood obesity
Source: Epigenetics. 2026 Sep 25;21(1):2732477. doi: 10.1080/15592294.2026.2732477 (PMC13618840; doi:10.1080/15592294.2026.2732477)
Supplement: Supplementary file 1 [file KEPI_A_2732477_SM2126.docx]

**Supplementary Figures**

**Supplementary Figure S1.** Flowchart of data filtering and child body composition categorization.

**Supplementary Figure S2.** Scatterplots of correlation between KM regression -log10(p-values) and PC regression -log10(p-values) for the unstratified and stratified analyses. Stratified plots are for the sustained obesity versus normal weight groups. Vertical and horizontal gray lines mark -log10(p-value) of 0.05 for KM regression on the x-axis and PC regression on the y-axis.

**a**

**b**

**Supplementary Figure S3.** Manhattan plot from CpG-level logistic regression stratified by maternal pre-pregnancy obesity status: (a) children of mothers with pre-pregnancy obesity and (b) children of mothers without pre-pregnancy obesity. In panel (a) the 20 most significant CpGs (ranked by raw p-value) within ICRs associated with sustained childhood obesity are highlighted in yellow and labeled with their nearest gene. No CpGs are lighted or labeled in panel (b) because no ICRs were associated with sustained obesity in this subgroup. The horizontal gray dotted line corresponds to a raw p-value of 0.05.

**a**

**b**

**Supplementary Figure S4.** Manhattan plot from CpG-level logistic regression stratified by child sex: (a) females and (b) males. In panel (a) the 20 most significant CpGs (ranked by raw p-value) within ICRs associated with sustained childhood obesity are highlighted in yellow and labeled with their nearest gene. No CpGs are lighted or labeled in panel (b) because no ICRs were associated with sustained obesity in this subgroup. The horizontal gray dotted line corresponds to a raw p-value of 0.05.
